# Aberrant structural covariance networks in youth at high familial risk for mood disorder

**DOI:** 10.1111/bdi.12868

**Published:** 2019-11-23

**Authors:** Kareen Heinze, Xueyi Shen, Emma Hawkins, Mathew A. Harris, Laura de Nooij, Andrew M. McIntosh, Stephen J. Wood, Heather C. Whalley

**Affiliations:** ^1^ School of Psychology University of Birmingham Birmingham UK; ^2^ Institute for Mental Health University of Birmingham Birmingham UK; ^3^ Centre for Human Brain Health University of Birmingham Birmingham UK; ^4^ Division of Psychiatry University of Edinburgh Edinburgh UK; ^5^ Orygen, The National Centre of Excellence in Youth Mental Health Melbourne Vic. Australia; ^6^ Centre for Youth Mental Health University of Melbourne Melbourne Vic. Australia

**Keywords:** bipolar disorder, executive control network, major depressive disorder, salience network, structural connectivity, structural imaging

## Abstract

**Objectives:**

Current research suggests significant disruptions in *functional* brain networks in individuals with mood disorder, and in those at familial risk. Studies of *structural* brain networks provide important insights into synchronized maturational change but have received less attention. We aimed to investigate developmental relationships of large‐scale brain networks in mood disorder using structural covariance (SC) analyses.

**Methods:**

We conducted SC analysis of baseline structural imaging data from 121 at the time of scanning unaffected high risk (HR) individuals (29 later developed mood disorder after a median time of 4.95 years), and 89 healthy controls (C‐well) with no familial risk from the Scottish Bipolar Family Study (age 15‐27, 64% female). Voxel‐wise analyses of covariance were conducted to compare the associations between each seed region in visual, auditory, motor, speech, semantic, executive‐control, salience and default‐mode networks and the whole brain signal. SC maps were compared for (a) HR(all) versus C‐well individuals, and (b) between those who remained well (HR‐well), versus those who subsequently developed mood disorder (HR‐MD), and C‐well.

**Results:**

There were no significant differences between HR(all) and C‐well individuals. On splitting the HR group based on subsequent clinical outcome, the HR‐MD group however displayed greater baseline SC in the salience and executive‐control network, and HR‐well individuals showed less SC in the salience network, compared to C‐well, respectively (*P* < .001).

**Conclusions:**

These findings indicate differences in network‐level inter‐regional relationships, especially within the salience network, which precede onset of mood disorder in those at familial risk.

## 1 INTRODUCTION

Bipolar disorder (BD) is a highly heritable affective disorder characterized by intense fluctuations in mood, and is one of the leading causes of disability worldwide.^1^ BD is associated with a variety of functional abnormalities, eg in cognitive control and affective networks. However, it is unclear whether these differences are pre‐existing and genetically mediated, or the consequence of differential maturation and divergent neurodevelopmental processes.^2^ One way to circumvent confounds such as medication and secondary disease abnormalities, is by looking into unaffected relatives, eg as done by the Scottish Bipolar Family Study^3^ and other familial high risk studies in mood disorder.^4^, ^5^, ^6^ Exploring anatomical relationships at the brain network level may uncover important insights about underlying disease mechanisms^2^ and may reveal neurobiological markers of risk and resilience, to inform effective prevention strategies.

The cerebral cortex is organised into complex large‐scale neurocognitive networks with reciprocal connections.^7^ Analyses of structural covariance (SC) offers insights into how inter‐individual differences in brain structure co‐vary with differences in other brain regions and notably show a reproducible organisation at the population level.^2^ The source of this variation of covariance patterns is not fully understood, but it has been demonstrated that SC networks have a strong genetic component,^8^ show robust and anatomically plausible differences across development^9^ as well as through the course of disease.^10^ Considering that adolescence and early adulthood are a critical period for co‐ordinated neurodevelopment and the onset of psychiatric disorders,^11^ aberrant development of structural covariance networks might contribute to the pathophysiology of mood disorders. Our group previously demonstrated subtle but significant changes in structural brain networks prior to psychosis onset in ultra‐high risk individuals using this approach.^12^ While in adult depression there have been indications of decreased SC in the salience network (SN)^13^ and default‐mode network (DMN), and higher levels of SC in the emotion regulation network versus controls,^14^ it is unclear, however, if these are present prior to onset of disorder.

Functional connectivity studies in individuals at high risk (HR) for mood disorder has revealed resting‐state connectivity differences, but with inconsistent directions^15^: some studies report less connectivity, eg of the inferior frontal gyrus^16^ and fronto‐occipital^17^ and anterior default‐mode^18^ network, others showed greater connectivity in the sensori‐motor^17^ and executive control network (ECN).^19^ Structural imaging studies show similarly inconsistent results with greater^20^ or less prefrontal gray matter volume,^21^ and less inferior frontal gyrus and insula white matter integrity^16^ in HR individuals compared to healthy controls (C‐well). Overall, there is accumulating evidence for neurobiological trait markers of risk^22^ and emerging evidence for connectivity increases between frontal brain areas as markers of resilience,^23^ however, it is unknown if differential structural alterations are underlying functional differences and whether they can provide more insight and consistency.

To our knowledge, this is the first study to explore *structural brain network architecture* in individuals at familial risk for mood disorder. The aim of the present study was to explore the evidence of neuroimaging markers of risk and resilience on the network level to identify markers of genetic predisposition, markers of early transition to an ill state, and adaptive responses associated with resilience. In the absence of evidence on structural brain networks in individuals at familial risk for mood disorder, we considered alterations in a number of resting‐state functional connectivity networks,^17^, ^18^, ^19^ and hypothesized that individuals at HR for mood disorder would exhibit different SC in large‐scale networks such as the SN, ECN, and DMN as compared to controls. Considering connectivity increases between frontal brain areas as markers of resilience,^23^ we expected that greater SC in the ECN would characterize individuals who remained well over the follow‐up period.

## 2 MATERIALS AND METHODS

### 2.1 Participants

121 individuals at familial risk for mood disorder—HR(all)—and 89 C‐well were recruited for the current study as part of the Scottish Bipolar Family Study. HR(all) individuals were identified via family members who had a diagnosis of bipolar 1 disorder who in turn were identified by psychiatrists across Scotland. Recruitment of the HR(all) group has been described in full previously.^3^, ^24^ Unaffected individuals who had one first‐ or two second‐degree relatives diagnosed with bipolar 1 disorder were invited to take part in the study. Unrelated, age‐, gender‐ and intelligence quotient‐matched controls without personal or family history of BD were identified from the social circle of the HR group. All participants were aged between 15‐27 years old. Both groups were screened using the Structured Clinical Interview for DSM‐IV‐TR Axis I Disorders (SCID)^25^ by two experienced psychiatrists (AMM, JES).

Individuals with a personal history of any major neurological or any DSM psychiatric diagnosis, learning disability, history of substance dependence, prior head injury that resulted in loss of consciousness, or any contraindication for magnetic resonance imaging (MRI) were excluded prior to baseline assessment. All participants were psychotropic medication‐naïve at baseline. There were n = 9 C‐well participants who were excluded from subsequent analyses due to development of mood disorder over the follow‐up. None of the remaining C‐well individuals developed any other psychopathology over the follow‐up period. Over the follow‐up period it was also discovered that one HR‐well participant had been diagnosed with a single episode of psychosis, but did not meet criteria for a psychotic disorder, or a mood disorder. Results excluding this participant are presented in Supporting Information. Lastly, one HR‐MD participant also had a diagnosis of emotional‐unstable personality disorder along with a mood disorder. Written informed consent was obtained from all participants and the study was approved by the Multi‐Centre Research Ethics Committee for Scotland.

All participants underwent an MRI scan and clinical assessment at baseline when all were well, and were clinically followed up over a median time of 4.95 years (range 3.3‐6.8 years). In total, 29 HR individuals were subsequently identified as having developed an affective disorder (HR‐MD, 27 developed MDD and two BD), whereas 92 remained well (HR‐well). Diagnostic status at follow‐up was determined either by face‐to‐face assessments (AMM, JES, and trained research assistants TS and AM) or through accessing National Health Service clinical records.

Manic, depressive and psychotic symptoms were rated using the Young Mania Rating Scales (YMRS),^26^ Hamilton Depression Rating Scale (HDRS)^27^ and Positive and Negative Syndrome Scale (PANSS),^28^ respectively.

### 2.2 Data acquisition and pre‐processing

T1‐weighted images were collected on a 1.5T GE Signa Horizon HDX (General Electric) clinical scanner equipped with a self‐shielding gradient set (22 mT/m maximum gradient strength) and manufacturer‐supplied “birdcage” quadrature head coil (time of inversion 500 ms, echo time 4 ms, flip angle 8°, voxel size 1.25 mm × 1.25 mm × 1.20 mm, 192 × 192 voxels, 180 slices).

We followed an a priori analytical plan based on our previous publication in ultra‐high risk for psychosis for pre‐processing^12^ and data analysis, including choice and analysis of seed regions.^9^ Images were manually reoriented and centred on the anterior commissure and normalised into standard space and segmented into gray matter, white matter and cerebrospinal fluid using a VBM8‐toolbox (http://dbm.neuro.uni-jena.de/spm) in SPM8 (Friston, The Welcome Department of Cognitive Neurology; http://www.fil.ion.ucl.ac.uk/spm) running in Matlab V7.9.0 (The MathWorks). The VBM8 toolbox used a unified segmentation approach that integrates tissue classification, image registration and inhomogeneity correction.^29^ The resulting segments were then smoothed using an 8‐mm full‐width at half‐maximum Gaussian kernel, to improve spatial resolution of the analyses. To study network SC, gray matter densities were derived using a standard 4‐mm‐radius spherical seed region of interest (ROI) chosen in accordance with and defined using the MarsBaR toolbox (http://marsbar.sourceforge.net) in SPM8.^30^ Seed regions were bilaterally defined in accordance with Zielinski et al^9^ in the visual (primary visual cortex, calcarine sulcus), auditory (primary auditory cortex, Heschl's gyrus), motor (primary motor cortex, precentral gyrus), speech (inferior frontal gyrus, pars opercularis), semantic (temporal pole), salience (fronto‐insular cortex), executive‐control (dorsolateral prefrontal cortex), and default‐mode (angular gyrus) networks (see Table S1).

In SPM8, we initially determined whole‐brain patterns of seed‐based structural covariance using individuals' grey matter volume maps for each seed in both hemispheres as covariate of interest in each group separately and used Threshold‐Free Cluster Enhancement (TFCE). Without being reliant upon hard threshold‐based clustering, this method optimizes areas of signal that show spatial contiguity. An algorithm runs though the image, with the aim to better distinguish between signal and noise.^31^ After employing the TFCE inference algorithm, the statistical threshold for the resulting correlation maps was set to *P* < .001 corrected for multiple comparisons using family‐wise error (FWE)‐correction at the whole‐brain level. Further, analyses of covariance (ANCOVAs) were performed for each seed region in both hemispheres. Considering the low spatial specificity of large clusters after using cluster‐extent based thresholding methods,^32^ we considered ANCOVA results significant at *P* < .001, FWE‐corrected at the whole‐brain level, after using TFCE. In addition to including mean gray matter volume of each seed region as covariate of interest, we included global gray matter and age as covariates of no interest in all analyses. Pairwise comparisons of covariance maps between grey matter volume of each seed voxel with grey matter volume of each voxel across the whole brain were reported for (a) HR(all) and C‐well, and (b) HR‐MD, HR‐well and C‐well. *P*‐values for the combined peak‐cluster level were Bonferroni‐corrected for multiple comparisons across eight networks in two hemispheres for the three‐group comparison (HR‐MD, HR‐well and C‐well; *P* = .05/48 = .00104).

Group differences concerning demographic and clinical data were determined using *t* and χ*^2^*‐squared tests for two‐group comparisons and Kruskal Wallis and Dunn‐Bonferroni post hoc tests for 3‐group comparisons using SPSS software, version 23.0 (http://www-01.ibm.com/software/analytics/spss/).

## 3 RESULTS

### 3.1 Demographics & description of sample

HR(all) individuals and C‐well did not differ significantly in terms of mean age (HR(all): M_age_ ± SD: 21 ± 2.9 years, range 15.2‐27.8; C‐well: M_age_ ± SD: 20.9 ± 2.4 years, range 16.3‐25.3; *t*_208_ = −0.275, *P* = .784), or gender (HR(all): 44 male/77 female; C‐well: 31 male/58 female; χ^2^_3_ = 0.052, *P* = .819). Likewise, HR‐MD (M_age_ ± SD: 20.9 ± 3.2 years, range 15.8‐27.8) and HR‐well (M_age_ ± SD: 21.1 ± 2.8 years, range 15.2‐26.2) individuals, did not differ significantly in terms of mean age (*t*_119_ = 0.18, *P* = .858), or gender (HR‐MD: 12 male/17 female; HR‐well: 32 male/60 female; χ*^2^*_3_ = 0.415, *P* = .52). Kruskal‐Wallis tests revealed group differences between HR‐MD, HR‐well and C‐well for HDRS, the PANSS positive and general subscale (see Table 1). Post‐hoc tests revealed significantly greater HDRS (*P* = .002), PANSS‐P (*P* = .011), and PANSS‐G (*P* = .001) in HR‐MD compared to C‐well. Further, PANSS‐G (were significantly higher in HR‐MD compared to HR‐well *P* = .011).

**Table 1:** Group differences for clinical measures at baseline

Measure	C‐well	HR‐well	HR‐MD	χ*^2^, P*
HDRS (n* *= 205)	0 ± 1	1 ± 2	1.5 ± 4	11.780, .003**
PANSS‐P (n* *= 207)	7 ± 0	7 ± 0	7 ± 1	9.254, .01*
PANSS‐N (n* *= 207)	7 ± 0	7 ± 0	7 ± 0	2.408, .3
PANSS‐G (n* *= 207)	16 ± 7	16 ± 2	18 ± 5	13.578, .001**
YMRS (n* *= 206)	0 ± 0	0 ± 0	0 ± 1	4.865, .088

Note

HR(all) = familial risk for mood disorder, C‐well = healthy control, HR‐well = high risk participants who did not develop mood disorder, HR‐MD = high risk participants who transitioned to mood disorder, HDRS = Hamilton Depression Rating Scale, PANSS = Positive and Negative Syndrome Scale (P = positive scale, N = negative scale, G = general psychopathology scale, YMRS = Young Mania Rating Scale, median ± interquartile range. *P < .05, **P < .01

### 3.2 Whole‐brain patterns of structural covariance in HR(all) and C‐well participants

**Figure 1:**
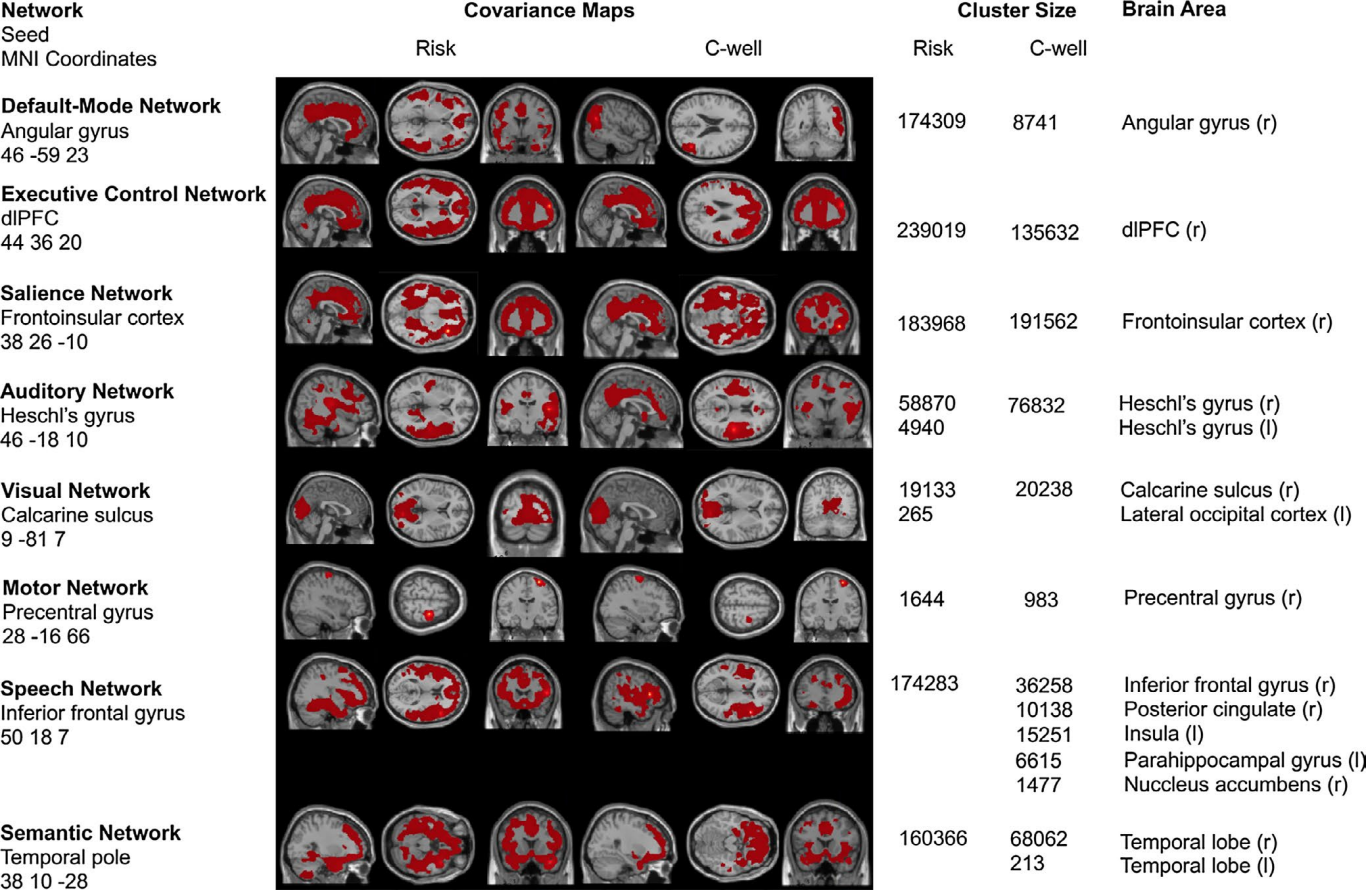
Patterns of structural covariance in familial high risk individuals compared to healthy control participants for the default‐mode, salience, executive‐control, visual, auditory, motor, speech, and semantic network (*P* < .001, family‐wise error corrected at whole brain and combined peak‐cluster level, threshold‐free cluster enhanced, clusters >100 continuous voxels reported). No significant group differences between HR(all) versus C‐well

Seed‐based SC mapping of the DMN, SN, ECN, visual, auditory, motor, speech and semantic network for both HR(all) and C‐well participants resembled standard canonical intrinsic connectivity^10^ and SC^9^ networks (Figure 1).

### 3.3 ANCOVAs comparing HR(all) and C‐well

There were no significant differences between HR(all) and C‐well individuals.

### 3.4 ANCOVAs comparing HR‐MD and HR‐well and C‐well

**Figure 2:**
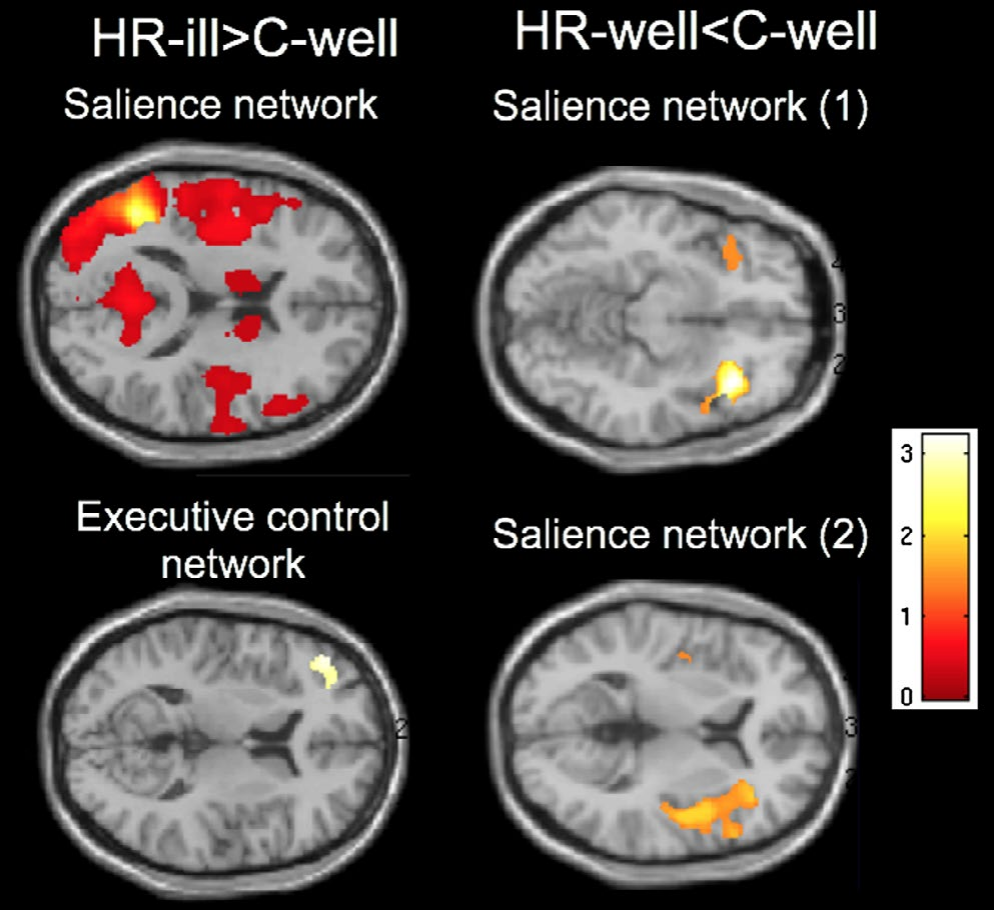
Axial slices of greater structural covariance (SC) in the salience network between the left fronto‐insular cortex and left angular gyrus, right insula, right thalamus and left temporal gyrus, and in the executive‐control network between the left dorsolateral prefrontal cortex and left inferior frontal gyrus in those who developed mood disorder as compared to controls; and less SC in the salience network between the right fronto‐insular cortex and the left and right orbitofrontal cortex, left inferior frontal gyrus and right insula in those who did not develop mood disorder as compared to C‐well. HR‐MD vs HR‐well: n/s. C‐well = healthy controls, HR‐MD = high risk participants who transitioned to mood disorder, HR‐well = high risk participants who did not develop mood disorder. *P* < .001, family‐wise error corrected at the whole‐brain level and *P* < .00104, family‐wise error‐corrected at the combined peak‐cluster level (clusters >10 consecutive voxels reported)

On separating the HR(all) group into subsequently ill/well, ANCOVAs revealed significantly greater SC for HR‐MD compared to C‐well in the seed for the SN between the left fronto‐insular cortex and left angular gyrus (k = 81 471, p_peak‐cluster/FWE_ < 0.001, −46 −58 22), right insula (k = 19 955, p_peak‐cluster/FWE_ < 0.001, 42 16 1), left thalamus (k = 2011, p_peak‐cluster/FWE_ = 0.001, −8 −3 13), and right temporal gyrus (k = 457, p_peak‐cluster/FWE_ = 0.001, 57 −43 −14). There was also greater SC in the seed for the ECN between the left dorsolateral prefrontal cortex and left inferior frontal gyrus (k = 1125, p_peak‐cluster/FWE_ < 0.001, −48 33 18) in HR‐MD individuals as compared to C‐well. Significantly less SC was found in the seed for the SN between the right fronto‐insular cortex and the right (k = 4786, p_peak‐cluster/FWE_ < 0.001, 38 22 −16) and left orbitofrontal cortex (k = −38, p_peak‐cluster/FWE_ = 0.001, −38 21 −18), right inferior frontal gyrus (k = 175, p_peakcluster/FWE_ = 0.001, 56 17 27) and left insula (k = 97, p_peakcluster/FWE_ = 0.001, −44 −10 7) in HR‐well individuals as compared to C‐well (Figure 2; Table 2). There were no statistically significant differences between HR‐MD and HR‐well individuals. Results were re‐analysed without the participant who developed a psychotic episode over the follow‐up period, and are presented in Table S2.

**Table 2:** Results from analyses of covariance (ANCOVA) for significant seed region covariance

Comparison	Network seed	Cluster size k (TFCE)	*P*‐value	MNI coordinates of cluster	Cluster classification
HR‐MD (n = 29) > C‐well (n = 89)	SN (l)	81 471	<.001***	−46 −58 22	Angular gyrus (l)
		19 955	<.001***	42 16 1	Insula (r)
		2011	.001**	−8 −3 13	Thalamus (l)
		457	.001**	57 −43 −14	Temporal gyrus (r)
	ECN (l)	1125	<.001***	−48 33 18	Inferior frontal gyrus (l)
HR‐well (n = 92) < C‐well (n = 89)	SN (r)	4786	<.001***	38 22 −11	Orbitofrontal cortex (r)
		633	<.001***	−38 21 −18	Orbitofrontal cortex (l)
		175	.001**	−44 −10 7	Inferior frontal gyrus (l)
		97	.001**	56 17 27	Insula (r)

Note

TFCE = threshold‐free cluster enhanced, MNI = Montreal Neurological Institute, C‐well = healthy controls, HR‐MD = high risk participants who transitioned to mood disorder, HR‐well = high risk participants who did not develop mood disorder, SN = salience network, ECN = executive‐control network, l = left, r = right, all clusters significant at *P* < .001, family‐wise error corrected at whole‐brain level, **P < .01, ***P < .001, family‐wise error corrected at combined peak/cluster level and adjusted for multiple comparisons for hemispheres (2), groups (3) and number of networks (8).

## 4 DISCUSSION

To our knowledge, this was the first study to compare network SC in individuals at familial risk for mood disorder. We found no significant differences in SC when comparing all individuals with a family history of mood disorder (all unaffected at baseline) and C‐well, and when comparing those at familial risk who developed mood disorder with those who did not over the follow up. However, there were distributed markers that differentiated those with familial risk who subsequently developed mood disorder over follow‐up and those who did not from C‐well, spanning across the SN and ECN and including areas such as the insula, angular, temporal, and inferior frontal gyrus and orbitofrontal cortex. Overall, we found *greater* inter‐connectivity between the SN and ECN for individuals at familial risk who developed mood disorder and *less* inter‐network connectivity for those who remained well over the follow‐up period, as compared to controls, respectively.

### 4.1 Familial risk

We found no significant differences in unaffected individuals at familial risk of mood disorder compared to controls. This is in contrast to a number of potential trait markers that have so far been identified for BD, eg widespread white matter reductions,^24^ thinning of temporal brain regions,^33^ and functional activation increases in the amygdala during task performance,^3^ but consistent with other recent studies that found no differences in functional resting‐state connectivity in individuals at familial risk for mood disorder,^34^ suggesting that these differences in connectivity may not be the most sensitive biomarkers of familial risk.

### 4.2 Markers to predict illness onset

Similarly, we did not find differences in SC between individuals at familial risk who developed mood disorder as compared those who did not. However, we did find diffuse differences in SC across the SN and ECN associated with subsequent onset of mood disorder, including in a variety of parietal, temporal, frontal and insular regions when compared to controls. While fronto‐temporal dysconnectivity and insula alterations have been repeatedly reported as trait markers for both BD and familial risk, they have been less commonly addressed as markers of early transition to an ill state. This is likely due to only few of these studies addressing biomarkers that may predict illness onset, and those that did, more specifically addressed emotional processing^35^ and focussed on areas relevant to emotion regulation, such as the amygdala.^36^

Nonetheless, our findings of greater SC in the SN and ECN are largely in line with altered pruning processes hypotheses in the inferior frontal and precentral gyrus^33^ and increased insula connectivity in those individuals who develop mood disorder.^37^ Where there is some evidence that SC networks mirror functional brain networks,^10^ SC networks are more likely to reflect synchronized maturational development.^38^ Even though SC analyses are not merely considered to provide a potential anatomical substrate for functional connectivity, our findings are consistent with the idea that development of structural, functional and maturational brain networks converge predominantly in frontal brain regions.^38^ However, longitudinal structural and functional connectivity studies are needed that stretch from child‐ to adulthood to address how structural and functional plasticity of cortical and subcortical brain areas and networks develop and interact over time.

Lastly, it is noteworthy to mention that individuals at familial risk for mood disorder, are not only at greater risk for developing BD or MDD but also other psychiatric disorders, such as schizophrenia.^39^ In our sample, one HR‐well participant experienced a single psychotic episode (who neither met criteria for schizophrenia, nor presented with evidence of mood disorder) and one HR‐MD participant has subsequently been diagnosed with emotional unstable personality disorder along with a mood disorder. We note that for analyses excluding this HR individual who experienced a single psychotic episode the main cluster between the right insula cortex and right orbitofrontal cortex remained significant.

### 4.3 Markers of non‐transition

Considering that the majority of HR individuals remain free of psychiatric pathology,^40^ the concept of resilience has recently been investigated (commonly in older HR individuals who are more likely to have passed the period of greatest risk).^23^ We found weaker inter‐network connectivity between the *SN* (insula) and ECN (orbitofrontal cortex) in those at increased familial risk who remained well as opposed to less SC in the ECN that we initially hypothesized. This finding is further contrasted by *greater* inter‐network connectivity between the SN (insula) and DMN (angular gyrus) in those who went on to develop mood disorder, as compared to C‐well that we discovered in the current study. Less SC between the insula and frontal regions may be interpreted as an adaptive response to maintain equilibrium by reducing the cognitive processing of potentially emotionally relevant environmental influences and internal responses. This may happen via an automatic redirection of attention away from emotionally salient stimuli, reducing excessive cognitive processing,^41^ and therefore limiting the vicious circle of increased emotional processing that is common to mood disorders.

Alternatively, findings could be explained with network control theory^42^: individuals at familial risk who develop mood disorder may have poor insula controllability, resulting in an over‐active DMN and ECN which is consistent with our findings of greater structural covariance of the SN seed with the angular gyrus and temporal lobe as part of the DMN and frontal brain regions in the ECN. Those at familial risk who remained well, may have a protective mechanism in place that homeostatically downregulates SN connectivity (even below control level), specifically with the ECN and frontal brain regions in our sample. This potential explanation is partly consistent with controllability deficits found in individuals at familial risk for mood disorder in prefrontal, superior temporal and striatal regions compared to controls.^43^

### 4.4 Limitations

A strength of this study is the longitudinal nature and follow‐up of five years, however, the diagnostic status of unaffected individuals at familial risk has nonetheless the potential to change^23^: whereas the majority of individuals will develop BD before the age of 25 years,^44^ peak age of mood disorders as a whole ranges between the late 20s to early 40s.^45^ Considering a mean age of 21 years at baseline and a five year follow‐up period, it is possible that a number of HR individuals are yet to develop mood disorder. We further have to acknowledge the low number of individuals who developed BD over the follow‐up and generally the cross‐over of risk for MDD and BD: a first episode of BD is often depressive in nature. Therefore, it is difficult to determine a definitive or stable diagnosis in this group of fairly young people. Secondly, we were not able to include all three groups for the group‐wise TFCE SC analyses in one model. To optimize sensitivity and spatial specificity and to ensure robustness of our findings, we used stringent and adjusted thresholds. Lastly, SC maps of the SN and ECN in adolescents and adults commonly display network parcellation overlap; consequently, testing for group differences may lack power and result in slightly deviated mapping for each network.

## 5 CONCLUSIONS

In conclusion, this study is the first to present a picture on network‐level SC in individuals at familial risk for mood disorder, and specifically address which cortical networks reflect risk and which could suggest resilience to the onset of mood disorder. Our findings prompt that markers of early transition to an ill state encompass widely distributed brain areas in the SN and ECN. Longer follow‐up periods are needed to ascertain whether individuals who have not transitioned over the five year period remain true negatives in the long‐term. With this in mind, our findings add to a number of imaging markers of risk and resilience that aim to inform precision medicine. Future studies with extended follow‐up periods will have the potential to ultimately aid clinical care by differentiating individuals who are likely to develop a mood disorder from those who are not.

## CONFLICT OF INTEREST

None.

## Data Availability

Data will be available upon reasonable request.
