# Supplementary material for: Aberrant structural covariance networks in youth at high familial risk for mood disorder
Source: Bipolar Disord. 2019 Nov 23;22(2):155–62. doi: 10.1111/bdi.12868 (PMC7155114; doi:10.1111/bdi.12868)
Supplement: Supplementary file 1 [file BDI-22-155-s001.docx]

Table S1

Coordinates for regions of interest in eight brain networks

| **Network** | **Seed Region** | **MNI Coordinates (x y z)** |
| --- | --- | --- |
| Default-Mode | Angular Gyrus | (-) 46 -59 23 |
| Salience  Executive Control  Visual  Auditory | Fronto-insular Cortex  Dorsolateral Prefrontal Cortex  Calcarine Sulcus  Heschl’s Gyrus | (-) 38 26 -10  (-) 44 36 20  (-) 9 -81 7  (-) 46 -18 10 |
| Motor | Precentral Gyrus | (-) 28 -16 66 |
| Speech | Inferior Frontal Gyrus | (-) 50 18 7 |
| Semantic | Temporal Pole | (-) 38 10 -28 |

*Notes*. MNI=Montreal Neurological Institute.

Table S2

*Results from analyses of covariance (ANCOVA) for significant seed region covariance*

*without the HR-well individual who developed psychosis*

| **Comparison** | **Network**  **Seed** | **Cluster size k**  **(TFCE)** | ***p*-value** | **MNI Coordinates**  **of cluster** | **Cluster classification** |
| --- | --- | --- | --- | --- | --- |
| HR-MD (*n=29*) >  C-well (*n=89*) | SN (l)  ECN (l) | 81471  19955  2011  457  1125  3136 | <0.001***  <0.001***  0.001**  0.001**  <0.001***  <0.001*** | -46 -58 22  42 16 1  -8 -3 13  57 -43 -14  -48 33 18  38 22 -11 | Angular gyrus (l)  Insula (r)  Thalamus (l)  Temporal gyrus (r)  Inferior frontal gyrus (l)  Orbitofrontal cortex (r) |
| HR-well (*n=92*) < C-well (*n=89*) | SN (r) |  |  |  |  |

*Notes.* TFCE=threshold-free cluster enhanced, MNI=Montreal Neurological Institute, C-well=healthy controls, HR-MD=high risk participants who transitioned to mood disorder, HR-well=high risk participants who did not develop mood disorder, SN=salience network, ECN=executive-control network, l=left, r=right, all clusters significant at p<0.001, family-wise error corrected at whole-brain level, ***p*<0.01, ****p*<0.001, family-wise error corrected at combined peak/cluster level and adjusted for multiple comparisons for hemispheres (2), groups (3) and number of networks (8).
